# Olaparib Combined With Dacomitinib in Osimertinib-Resistant Brain and Leptomeningeal Metastases From Non-Small Cell Lung Cancer: A Case Report and Systematic Review

**DOI:** 10.3389/fonc.2022.877279

**Published:** 2022-04-14

**Authors:** Hui Zhang, Yong Wang, Huaguo Wu, Shizhen Zhou, Shuo Li, Xiangji Meng, Rongjie Tao, Jinming Yu

**Affiliations:** ^1^ Tianjin Cancer Institute, Key Laboratory of Cancer Prevention and Therapy, National Clinical Research Center for Cancer, Tianjin Medical University Cancer Institute and Hospital, Tianjin’s Clinical Research Center for Cancer, Tianjin Medical University, Tianjin, China; ^2^ Department of Medical Oncology, Shandong Cancer Hospital and Institute, Shandong First Medical University and Shandong Academy of Medical Science, Jinan, China; ^3^ Department of Neurosurgery, Shandong Cancer Hospital and Institute, Shandong First Medical University and Shandong Academy of Medical Science, Jinan, China; ^4^ Department of Head and Neck Surgery, Shandong Cancer Hospital and Institute, Shandong First Medical University and Shandong Academy of Medical Science, Jinan, China; ^5^ School of Medicine and Life Science, University of Jinan-Shandong Academy of Medical Science, Jinan, China; ^6^ Department of Radiation Oncology, Shandong Cancer Hospital and Institute, Shandong First Medical University and Shandong Academy of Medical Science, Jinan, China

**Keywords:** olaparib, dacomitinib, osimertinib-resistant, brain and leptomeningeal metastases, non-small cell lung cancer

## Abstract

Lung cancer patients with brain and leptomeningeal metastases usually have poor prognosis. For those patients with EGFR mutations, osimertinib, a third-generation tyrosine kinase inhibitor (TKI), is the first choice of treatment. However, drug resistance to osimertinib frequently occurs; and to date, the available follow-up treatment strategies have limited efficacy. In this case study, we report that treatments with olaparib, a Poly (ADP-ribose) polymerase (PARP) inhibitor, combined with dacomitinib, a second-generation EGFR TKI, benefited a lung cancer patient with osimertinib-resistant brain and leptomeningeal metastases. This 55-year-old male patient was found to have a pL858R mutation on EGFR exon 21 combined with TP53 and ERBB2 mutations after developing drug resistance to osimertinib treatment. Based on the genetic testing results, he was treated with olaparib and dacomitinib, and obtained 6 months of progression-free survival (PFS) and 13 months of overall survival (OS) after the diagnosis of leptomeningeal metastasis. This case report represents the first study applying PARP inhibitor in combination with dacomitinib in the treatment of leptomeningeal metastases after osimertinib resistance.

## Introduction

Patients with central nervous system (CNS) metastases from non-small cell lung cancer (NSCLC) usually have very poor prognosis (1, 2). The median survival of patients in this population is usually less than 1 year despite the emerging advancement in treatment options (3–5). It has been reported that in patients with NSCLC, up to 50% would develop brain metastases (BM) during the course of their illness (5) and 3-5% would develop leptomeningeal metastases (LM) (6). The incidence could be higher in those with anaplastic lymphoma kinase (ALK)-rearrangement or epidermal growth factor receptor (EGFR) mutations (7). Currently, a combinational strategy of multidisciplinary therapies involving systemic and intrathecal chemotherapy, radiotherapy, and targeted therapies (such as osimertinib and bevacizumab) is preferred for NSCLC patients with brain and leptomeningeal metastases (2, 8). However, the reported survival time remains unsatisfied and more investigation on treatment strategies is needed.

Osimertinib (AZD9291), a third-generation oral irreversible EGFR tyrosine kinase inhibitor (TKI) (9), has been approved by the United States Food and Drug Administration (FDA) and the European Medicines Agency (EMA) for patients with acquired EGFR T790M mutation (10). In preclinical studies, osimertinib exhibited greater penetration of the mouse blood-brain barrier than gefitinib, rociletinib, or afatinib at clinically relevant doses and showed some effectiveness for treating the first or second-generation EGFR-TKI resistant leptomeningeal metastases from EGFR-mutant lung cancer (11, 12). In clinical studies, osimertinib also showed significant intracranial activity (13, 14). In the most recent prospective phase II study which evaluated the efficacy of osimertinib 160 mg in T790M-positive BM or LM of NSCLC patients who progressed on prior EGFR TKI treatment, the median overall survival was 16.9 months in the BM cohort and 13.3 months in the LM cohort (14).

In spite of the success of osimertinib as a therapy for patients with NSCLC and its potential efficacy for CNS metastases, acquired resistance involving EGFR-dependent or EGFR-independent mechanisms inevitably occurs and hampers its clinical benefits (15). It is estimated that when osimertinib is used as a first-line treatment, resistance develops approximately after 19 months of treatment; and when it is used as a second-line treatment, resistance could occur after 11 months (16). The mechanisms of resistance to osimertinib are complicated, but most of them involved in EGFR exon 20 mutations (C797S, M766Q, S768I, L718 V, and others) leading to disruption of osimertinib binding sites, alternative pathway activation, aberrant downstream signaling and lineage plasticity leading to small cell transformation, such as *MET* and *ERBB2* amplifications, inactivation of *TP53* and/or *RB1*, and so on (15, 16). The good treatment option for patients who develop resistance to osimertinib remains a critical unresolved issue in the field. Current clinical trials are focusing on targeting alternative pathways (resistance mediated by *MET*, *ERBB2*, and C979S mutation) and combination of VEGF inhibitions with EGFR-TKIs (clinical trials: NCT03392246, NCT03784599, NCT04181060, NCT03909334, and so on.) (16, 17).

Poly (ADP-ribose) polymerase 1 (PARP1) is an important DNA repair enzyme of the base excision repair (BER) pathway and represents a critical target in cancer treatments (18). FDA has approved four PARP inhibitors (Olaparib, Rucaparib, Niraparib, and Talazoparib) for treatments of ovarian and breast cancers (19). To date, very few studies have reported their efficacies in lung adenocarcinoma. A pre-clinical study by Lynnette Marcar et al. showed that compared to TKI sensitive cells, TKI (gefitinib and osimertinib) resistant EGFR mutant NSCLC cells were more sensitive to PARP inhibitors, indicating the potential efficacy of PARP inhibitors in treating osimertinib-resistant NSCLC (20). A phase I clinical trial investigating the efficacy of the PARP inhibitor, niraparib, together with osimertinib in treating patients with stage IV EGFR-mutated NSCLC is ongoing (NCT03891615).

In this case study, we report that the treatment using PARP inhibitor olaparib in combination with dacomitinib, a second generation EGFR-TKI, benefited a NSCLC patient with osimertinib-resistant brain and leptomeningeal metastases who had EGFR mutations combined with TP53 and ERBB2 mutations.

## Case Presentation

**Figure 1:**
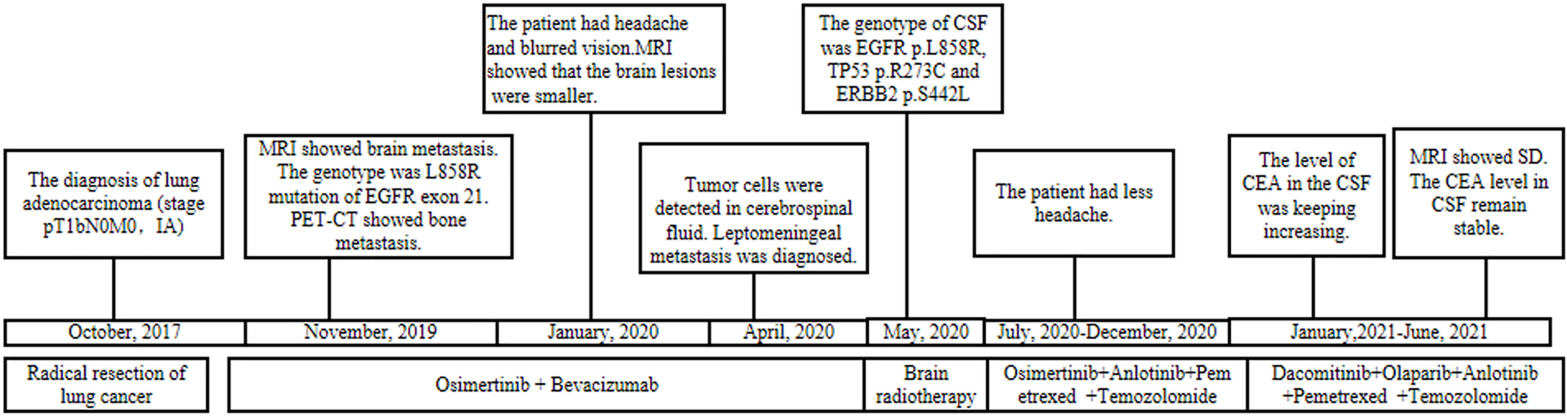
Summary of the diagnosis and treatment process.

**Figure 2:**
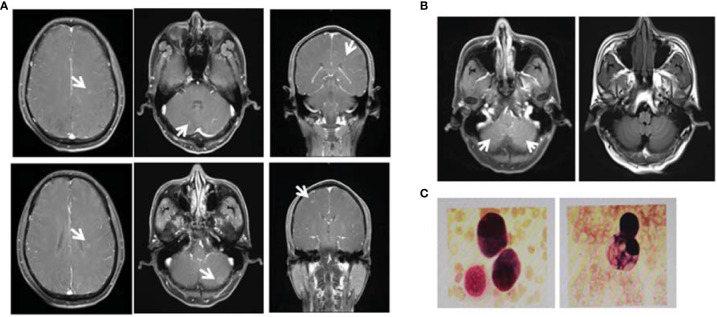
Diagnosis of brain metastases. **(A)** On October 31, 2019, the magnetic resonance imaging (MRI) examination of the patient showed multiple brain masses including multiple metastases in the lateral ventricle, right frontal lobe, and cerebellar hemisphere. Diagnosis of leptomeningeal metastases. **(B)** On April 21, 2020, MRI T1 enhancement and FLARE examination showed that the cerebellar sulci had multiple line-like and spot-like enhancements, suggesting meningeal metastasis. **(C)** On April 30, 2020, retest of the cerebrospinal fluid (CSF) showed the presence of cancer cells with the characteristics of adenocarcinoma cells.

**Figure 3:**
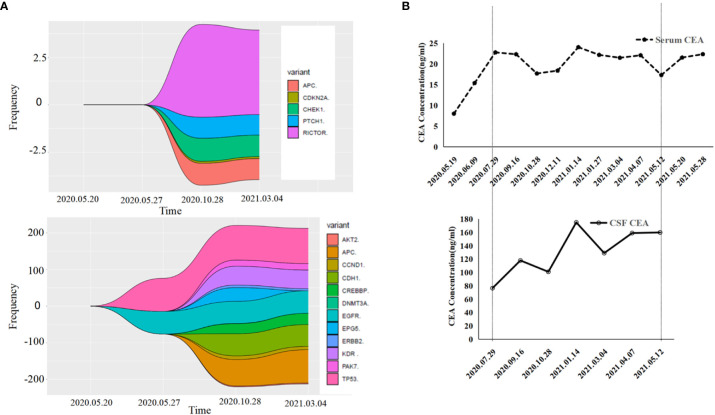
Gene detection diagram and levels of carcinoembryonic antigen (CEA) during treatment. **(A)** Gene detection diagram during the treatment was shown. Different colors represent different genes and different wave widths. The number on the vertical axis represents the mutation frequency (wave width). **(B)** The levels of CEA, including serum CEA and cerebrospinal fluid CEA, were shown.

**Figure 4:**
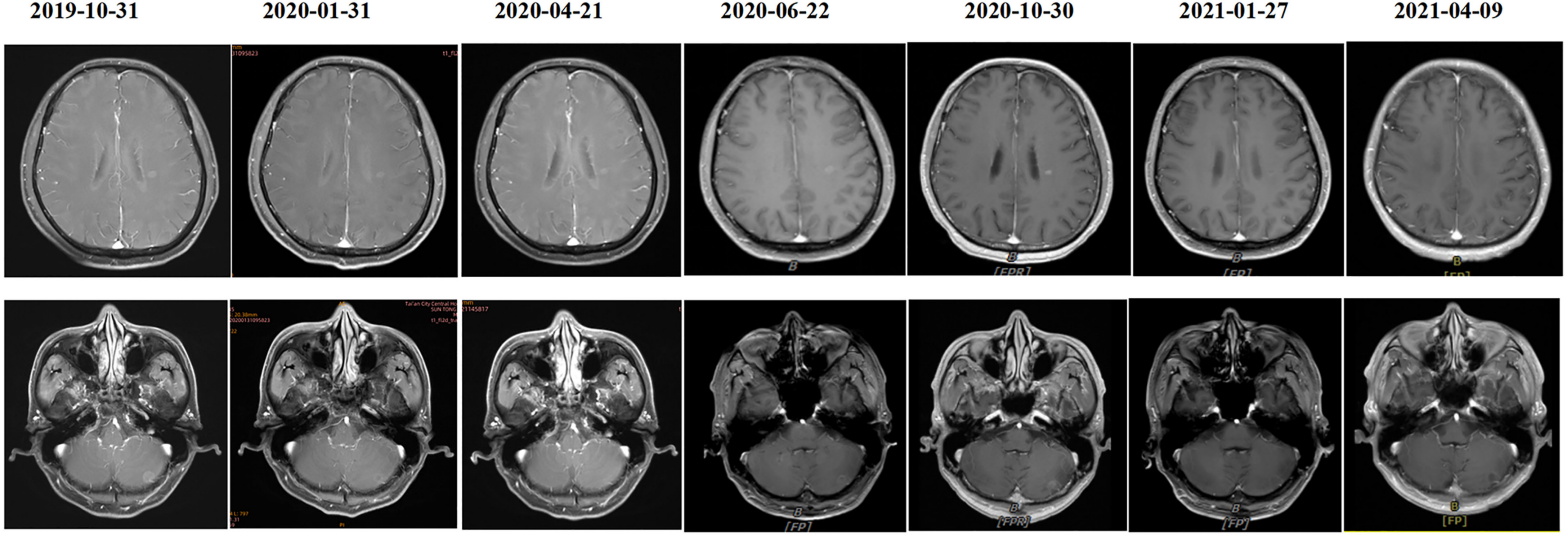
MRI imaging changes at different time points during the treatment process.

A 55-year-old male underwent thoracoscopic-assisted small-incision radical resection of lung cancer in October 11, 2017 after diagnosis of lung adenocarcinoma (stage pT1bN0M0, IA) (**Figure 1**). His postoperative regular magnetic resonance imaging (MRI) and computerized tomography (CT) examinations showed no obvious abnormalities. Two years later (on October 12, 2019), the patient started to experience headache, blurred vision, and hearing loss. On October 31, 2019, his brain MRI exam showed multiple brain masses including multiple metastases in the lateral ventricle, right frontal lobe, and cerebellar hemisphere (**Figure 2A**). His genetic testing revealed the L858R mutation in exon 21 of EGFR. His positron emission tomography (PET) CT scan suggested bone metastasis (**Figure S1**). The patient was then treated with osimertinib (80 mg oral administration daily), bevacizumab (7.5mg/kg q3w), and bisphosphonate (4mg q3w). After treatment for 1 month, his symptom of headache almost disappeared, but he developed blurred vision and unstable walking on both feet. On 2020-1-4, the patient complained increased blurred vision and occasional headache. On 2020-4-21, his brain MRI (**Figure 2B**) showed multiple dot-line enhancements (masses) in the cerebellar sulci, suggesting meningeal metastasis. On 2020-4-30, the patient underwent cerebrospinal fluid (CSF) cytology examination and cancer cells were found in the cerebrospinal fluid, which was in line with the characteristics of adenocarcinoma cells (**Figure 2C**). His CSF genetic testing results in May 2020 showed that mutations were EGFR p.L858R, TP53 p.R273C and ERBB2 p.S442L (**Figure 3A**). The patient was then treated with whole brain radiotherapy (40Gy/20 fractions). Between July 2020 and December 2020, the patient was treated with osimertinib (80 mg oral administration daily), anlotinib (12mg d1-14, q3w), pemetrexed (500mg/kg q4w), and temozolomide (300mg d1-5, q4w). Then, the patient experienced less headache. The re-test of the CSF samples showed that the mutations included EGFR p.L858R, TP53, ERBB2, and others (October 28, 2021). (**Figure 3A**). In January 2021, due to the continued elevated levels of tumor marker carcinoembryonic antigen (CEA) in his CSF (**Figure 3B**) and based on his CSF genetic test results, his treatment was switched to dacomitinib (30mg po qd), olaparib (100mg po bid), anlotinib (8mg po d1-14,q3w), pemetrexed (500mg/m^2^, q3w), and temozolomide (200mg/m^2^ d1-5, q4w). The patient’s condition was stable after adjusting the treatment plan. His level of CEA was stable (**Figure 3B**), and his brain MRI showed no progression of the brain metastasis. The efficacy evaluation during the treatment period is shown in **Figure 4**. In addition, after switching to targeted therapy, the patient no longer experienced obvious headache and dizziness, and his quality of life was significantly improved. Although he had the 2nd degree bone marrow suppression, there was no obvious functional damage of the liver and kidney during the process of treatment. The patient died of cachexia caused by the tumor in June 2021. Based upon, targeted drug therapy helped this patient obtain 6 months of progression-free survival (PFS). After the diagnosis of CNS metastases, although he was no longer sensitive to osimertinib, his overall survival reached 13 months which might be benefited from the treatment of olaparib in combination with dacomitinib.

## Discussion

This case represents the first study applying PARP inhibitor combined with dacomitinib for treating CNS metastases from NSCLC after osimertinib resistance. Using this new treatment strategy in combination with other chemotherapeutic drugs and radiotherapy, the patient eventually obtained 6 months of progression-free survival and 13 months of overall survival after the diagnosis of leptomeningeal metastasis, which is markedly longer than the median survival reported in previous studies (3, 4, 21).

Unlike a previous case report which showed that dacomitinib, the second-generation TKI, did not benefit a patient who developed mutations after later-line osimertinib treatment (22), the present case with ERBB2 mutations showed a different outcome. This patient also had mutations in TP53, a gene that is essential for DNA repair. Given the effects of PARP inhibitors in DNA repair pathway, this patient was treated with olaparib and seemed to be benefited from this combined targeted therapy.

This case report also highlighted the importance of CSF genetic testing in the diagnoses of CNS metastases. For this patient, his plasma genetic testing in May 2020 was negative for EGFR mutations; however, his CSF samples were positive and showed mutations in EGFR p.L858R, TP53, and ERBB2, which helped determining his subsequent treatment strategies (dacomitinib and olaparib). This case suggests that CSF genetic testing is necessary for patients who may have CNS metastases. Indeed, CSF-derived cell free DNA holds promise for diagnosis and characterization of CNS tumors or metastases (23). In a study involving 26 lung cancer patients with leptomeningeal metastasis and known EGFR mutations in the primary tumor, mutations in driver genes were detected in 100% (26/26), 84.6% (22/26), and 73.1% (19/26) of samples from CSF cell-free DNA (cfDNA), CSF precipitates, and plasma, respectively (24). Therefore, genetic profiling of CSF samples could help the diagnoses of patients with CNS metastases and improve their treatment outcomes.

For cancer patients, a significant increase in CEA level is often related to meningeal metastases (25). Therefore, CEA levels in CSF samples could be an indicator for the prognosis. In this case, the CEA levels in the CSF samples were notably higher than that in the blood. After targeted therapy, his CEA levels were stable, which was consistent with his MRI test results.

CNS metastases frequently occur in patients with lung cancer; and these patients often have an extremely poor prognosis. Due to the poor penetration of the blood-brain barrier, the first- and second- generation of EGFR-TKIs have low efficacy in the treatment of BM and LM in NSCLC. Although the third generation of EGFR-TKI, osimertinib, has shown increased penetration into the blood-brain barrier and enhanced clinical activity in patients with CNS metastasis, unfortunately, similar to the first- and second- generation of EGFR-TKIs, drug resistance to osimertinib commonly occurs after over 1 year of treatment. As reviewed by Alessandro Leonetti et al., the known mechanisms of resistance to osimertinib include EGFR modifications, activation of bypass signaling pathways mediated by MET, ERBB2, ALK, IGFR1, FGFR, and others, downstream pathway activation, epithelial-to-mesenchymal transition, histologic transformation, oncogenic gene fusions and cell-cycle gene aberrations (15).

Currently, platinum-based chemotherapy remains the standard of care after osimertinib resistance develops. Several early phase clinical trials for overcoming osimertinib resistance in NSCLC patients are ongoing. The strategies in these trials are mainly targeting specific known resistance mechanisms, including MET-inhibitors, MEK inhibitors, or combination of VEGF-inhibition with EGFR-TKIs (16). According to the phase Ib TATTON study involving patients with advanced EGFR-mutant NSCLC, osimertinib with either the MEK1/2 inhibitors (selumetinib or durvalumab), MET-inhibitor (savolitinib), or the PDL1-inhibitor (durvalumab) seemed to be safe and tolerable by patients (26). Currently the phase II clinical trial testing osimertinib plus savolitinib in EGFRm+/MET+ NSCLC patients following prior osimertinib (SAVANNAH) is still ongoing (NCT03778229). In another phase II study recruiting patients with advanced NSCLC who progressed on first-line osimertinib therapy (ORCHARD; NCT03944772), up to 9 experimental modules are tested: including Osimertinib + Savolitinib, Osimertinib + Gefitinib, Osimertinib + Necitumumab, Carboplatin + Pemetrexed + Durvalumab, Osimertinib + Alectinib, Osimertinib + Selpercatinib, Etoposide + Durvalumab + Carboplatin or Cisplatin, Osimertinib + Pemetrexed + Carboplatin or Cisplatin, Osimertinib + Selumetinib. However, the estimated primary completion date is set to be in the year of 2025. Therefore, whether these strategies could be beneficial to NSCLC patients with CNS metastasis and osimertinib resistance remains unclear. Immunotherapy using immune checkpoint inhibitors has been utilized in EGFR-mutated NSCLC patients, though its efficacy seemed to be not as good as in other cancers (27). Currently it is not clear whether immunotherapy has beneficial effects in NSCLC patients with Osimertinib resistance. However, it should be noted that interstitial lung disease-like events could occur when combining checkpoint inhibitors with osimertinib according to the phase Ib TATTON trial which assessed treatment of osimertinib in combination with durvalumab (26).

This case report in the patient with mutations in EGFR pL858R, TP53, and ERBB2 supported that dacomitinib, a second-generation EGFR-TKI, together with olaparib, a PARP inhibitor, may benefit NSCLC patient with CNS metastases after developing osimertinib resistance. The possible mechanisms could be as followed. First, dacomitinib is a selective inhibitor for ERBB2. It has been reported that dacomitinib could suppress the proliferation of Ba/F3 cells expressing T790M in *cis* to different deletions in exon 19 (IC50: 140-330 nmol/L) (28). Since this patient was tested positive for ERBB2 mutation in his CSF samples, dacomitinib might be the most relevant drug and he obtained the beneficial treatment result. Second, as suggested in the *in vitro* and *in vivo* experiments by Lynnette Marcar et al., the PARP inhibitor could increase reactive oxygen species (ROS) production and induce oxidative damage in osimertinib-resistant EGFR mutant NSCLC cells (20). Third, in triple-negative breast cancer, it has been shown that TP53 mutations combined with BRCA1 mutations are very frequent and may be sensitive to PARP inhibitors (29). Therefore, PARP inhibitors may also benefit NSCLC patients with TP53 mutations, which requires further evaluation. Moreover, it is known that P-glycoprotein (P-gp) is one of the proteins expressed naturally on the plasma membrane of endothelial cells in the blood-brain barrier (BBB), that could restrict substrate compounds from entering the brain (30). PARP inhibitors including olaparib, veliparib, and CEP-8983 are P-glycoprotein substrates (31). Therefore, olaparib may help enhance the antagonizing effect of dacomitinib on multidrug resistance, by inhibiting the efflux activity of ABCB1 and ABCG2 transporters (32).

In summary, exploring treatment strategies for patients with acquired osimertinib drug resistance is critical, particularly for patients with CNS metastases. The cerebrospinal fluid genetic testing plays an important role in guiding individualized treatment of patients with CNS metastases, as appropriate targeted treatments could be selected based on testing results. PARP inhibitors, olaparib, combined with EGFR-TKIs, may benefit patients with CNS metastases of NSCLC, and this combination strategy of multiple-target therapy is safe and holds promise for clinical application.

## Data Availability Statement

The datasets presented in this study can be found in online repositories. The names of the repository/repositories and accession number(s) can be found in the article/**Supplementary Material**.

## Ethics Statement

The studies involving human participants were reviewed and approved by Ethics Committee of Shandong Cancer Hospital and Institute. The patients/participants provided their written informed consent to participate in this study.

## Author Contributions

HZ, YW, XM, and SZ collected the clinical and pathological data. HW and HZ wrote the manuscript. JY and RT assisted in revising the manuscript. All authors contributed to the article and approved the submitted version.

## Funding

This work was supported by funds from the National Natural Sciences Foundation of China (no. 8150111724; to HZ), the Joint Fund for Cancer Prevention and Treatment of Shandong Natural Fund (no. ZR2019LZL015; to HZ), and Wu Jieping Medical Fund (no.320.6750.19088-24; to HZ).

## Conflict of Interest

The authors declare that the research was conducted in the absence of any commercial or financial relationships that could be construed as a potential conflict of interest.

## Publisher’s Note

All claims expressed in this article are solely those of the authors and do not necessarily represent those of their affiliated organizations, or those of the publisher, the editors and the reviewers. Any product that may be evaluated in this article, or claim that may be made by its manufacturer, is not guaranteed or endorsed by the publisher.
