# Supplementary material for: Olaparib Combined With Dacomitinib in Osimertinib-Resistant Brain and Leptomeningeal Metastases From Non-Small Cell Lung Cancer: A Case Report and Systematic Review
Source: Front Oncol. 2022 Apr 14;12:877279. doi: 10.3389/fonc.2022.877279 (PMC9047901; doi:10.3389/fonc.2022.877279)
Supplement: Supplementary file 1 [file DataSheet_1.docx]

Figuer s1


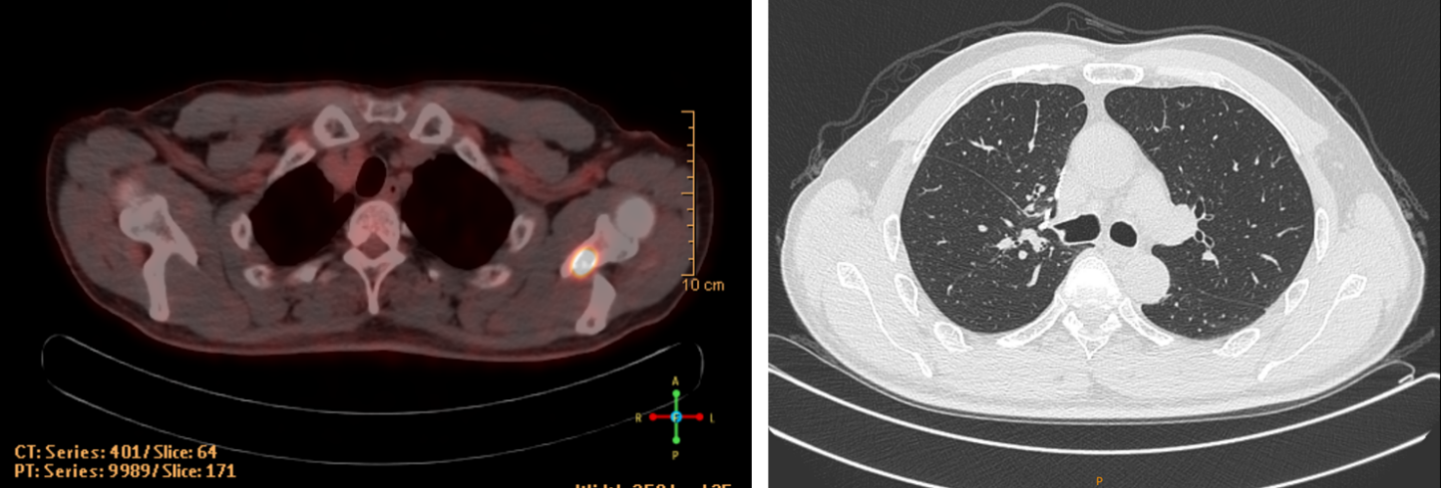


**Figure S1. PET-CT examination before treatments with olaparib and dacomitinib.** 2019-11-6 PET-CT examination showed focal osteolytic bone destruction of the left scapula with obvious high FDG metabolism, bone metastases, and stable chest lesions after lung cancer resection.
